# 1Novel *MEFV *transcripts in Familial Mediterranean fever patients and controls

**DOI:** 10.1186/1471-2350-11-87

**Published:** 2010-06-09

**Authors:** Myrna Medlej-Hashim, Nancy Nehme, Eliane Chouery, Nadine Jalkh, André Megarbane

**Affiliations:** 1 Unité de Génétique Médicale. Faculté de Médecine, Université Saint Joseph, Beirut, Lebanon; 2 Department of Life and Earth Sciences, Faculty of Sciences, Branch II, Lebanese University, Beirut, Lebanon

## Abstract

**Background:**

Familial Mediterranean fever is a recessive autoinflammatory disease frequently encountered in Armenians, Jews, Arabs and Turks. The *MEFV *gene is responsible for the disease. It encodes a protein called pyrin/marenostrin involved in the innate immune system. A large number of clinically diagnosed FMF patients carry only one *MEFV *mutation. This study aims at studying the *MEFV *gene splicing pattern in heterozygous FMF patients and healthy individuals, in an attempt to understand the mechanism underlying the disease in these patients.

**Methods:**

RNA was extracted from peripheral blood leucocytes of 41 FMF patients and 34 healthy individuals. RT-PCR was then performed, and the amplified products were migrated on a polyacrylamide electrophoresis gel, characterized by gel extraction of the corresponding bands followed by sequencing.

**Results:**

Five novel splicing events were observed in both patients and controls deleting either exons 3, 4 (del34), or exons 2, 3, 4 (del234), or exons 2, 3, 4, 5 (del2345) or exon7 (del7) or exons 7 and 8 (del78).

**Conclusions:**

The observation of such qualitative variability in the expression of the *MEFV *gene suggests a complex transcriptional regulation. However, the expression of these novel transcripts in both patients and controls is not in favour of a severe pathogenic effect.

## Background

Familial Mediterranean fever (FMF) is an autoinflammatory autosomal recessive disease particularly frequent around the Mediterranean basin. It is characterized by recurrent bouts of fever and serosal inflammation, the most severe manifestation of the disease being renal amyloidosis [1]. The *MEFV *gene on chromosome 16p13.3, responsible for this disease [2,3], is composed of 10 exons. Over 80 *MEFV *mutations were detected in FMF patients and registered in the autoinflammatory mutation database Infevers http://fmf.igh.cnrs.fr/infevers/[4]. Five of these mutations (M694V, M694I, V726A, M680I and E148Q) were mostly encountered in the mainly affected populations, namely Jews, Armenians, Arabs and Turks [5]. *MEFV *encodes a 781 amino acids' protein named pyrin or marenostrin (P/M) that is involved in the inflammatory pathways of the innate immune system [6]. Sequence alignment of the protein revealed 6 domains: a pyrin domain (or PyD), a bZIP basic domain, a B-box zinc finger domain, a coiled coil domain, 2 nuclear localization signals domains and a Ret Finger protein (RFP or B30.2) domain, also known as SPRY domain [7,8].

*MEFV *is expressed in neutrophils, eosinophils, monocytes [9,10] and to a lesser extent in skin and peritoneal fibroblasts [11]. Several alternatively spliced *MEFV *transcripts have been previously described. The first one (*MEFV*-d2) was identified in peripheral blood leukocytes (PBLs) and lacks exon 2 [12]. Diaz et al. characterized 3 other transcripts in synovial fibroblasts from osteoarthritis affected patients, one substituting exon 2a for exon 2 (2a), one with an extra exon corresponding to a sequence in intron 4 (4a), and one with an extension of exon 8 (8ext) [13]. Moreover, *MEFV*-d2 and 2a combined with 4a or 8ext to form four other transcripts: Δ2/4a, 2a/4a, Δ2/8ext and 2a/8ext. The various combinations of 4a and 8ext result in a frameshift leading to putative truncated proteins [13].

Clinical diagnosis is often confirmed by genetic testing of the *MEFV *gene. However, in an important number of clinically diagnosed FMF patients, only one *MEFV *mutation was detected by screening of the genomic *MEFV *coding sequence [5,14]. In the present study, we examined the splicing pattern of *MEFV *in normal and FMF PBLs, in an attempt to understand the mechanism underlying FMF in the clinically diagnosed patients carrying only one *MEFV *mutation. A qualitative analysis of *MEFV *transcripts was conducted in 41 FMF patients and 34 healthy individuals. Results were compared, and plotted to the patient genotype data. Our study did not show evidence for any correlation between genotypes and splicing, but revealed 5 novel transcripts which increases the number of transcripts identified in human leukocytes.

## Methods

### Human material from FMF patients and controls

This study included 2 series consisting of 41 unrelated Lebanese FMF patients who fulfilled international diagnostic FMF criteria [15] and 34 Lebanese healthy individuals. 36 of the FMF patients had only one identifiable exonic mutation and 5 patients had 2 mutations (Table 1). Written consent was obtained from all individuals, and the work was approved by the Saint Joseph University ethical committee. Blood was collected into tubes containing heparin, and whole PBLs were purified by lysis of the red cells with blood lysis buffer according to standard protocols.

**Table 1:** *MEFV *genotypes and splicing events in FMF patients and healthy controls.

Genotype	Number of patients	5' Splicing events^a^			3' Splicing events^a^	
		del34	del234	del2345	del7	del78
Genetically confirmed patients						
**p.[M694V]+[=]**	1		**+**		**+**	**+**
	1					
**p.[M694I]+[=]**	1				**+**	**+**
**p.[M680I]+[=]**	1	**+**	**+**		**+**	**+**
**p.[V726A] +[=]**	1		**+**	**+**		

Heterozygous patients						
**p.[M694V]+[?]**	4		**+**		**+**	**+**
	4					
	1		**+**			
	1				**+**	**+**
	1					
**p.[M694I]+[?]**	1		**+**		**+**	**+**
	1		**+**			
**p.[V726A] +[?]**	2	**+**				
	1					
**p.[E148Q] +[?]**	3					
	3				**+**	**+**
	2		**+**	**+**	**+**	**+**
	1	**+**	**+**		**+**	**+**
	1		**+**			
**p.[M680I]+[?]**	1					
	1		**+**		**+**	**+**
**p.[P369S/R408Q]+[?]**	2		**+**	**+**	**+**	**+**
	1		**+**		**+**	**+**
**p.[A744S]+[?]**	2					
**p.[K695R]+[?]**	2		**+**		**+**	**+**
**p.[R761H]+[?]**	1		**+**			

Healthy controls						
	11		**+**			
	9		**+**		**+**	**+**
	9					
	1		**+**	**+**		
	1				**+**	**+**
	1	**+**			**+**	**+**
	1		**+**	**+**	**+**	**+**
	1	**+**	**+**	**+**	**+**	**+**
						

^a ^The **+ **sign indicates the presence of the corresponding splicing event.

### RT-PCR analysis

Total RNAs were extracted from PBLs using the phenol chloroform method, then retro-transcribed (RT) into complementary DNA (cDNA) using random primers. Two sets of Polymerization Chain Reaction (PCR) were run on the *MEFV *cDNAs. The 5' amplicon was obtained with primers 5' AGCCAGATCCAGAGAGCCA 3' in exon 1 and 5' CCTGTGCAAGATGTCTCCAA 3' in exon 6 and the 3' amplicon with primers 5' TGCAGAGGAAGCTGGAGCA 3' in exon 5 and 5' ACCTCCACCTCCCAGTAACGG 3' in exon 10. The amplified products were migrated on a polyacrylamide gel and characterized by extraction of the corresponding bands from the gel then sequencing on an ABI Prism 3130 genetic analyzer.

## Results

**Figure 1:**
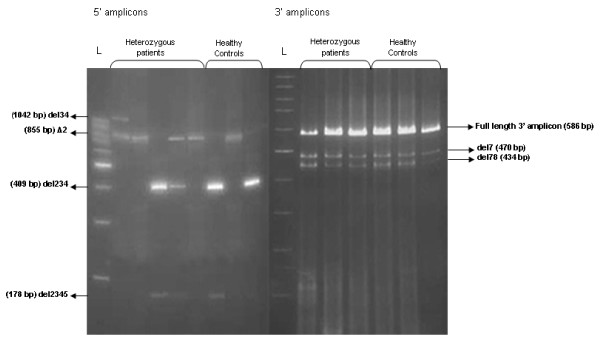
**Two representative RT-PCR electrophoresis gels showing the novel isoforms observed from the 5' amplified segment (left) and the 3' amplified segment (right) of the *MEFV *transcripts in heterozygous patients and healthy controls**. The 2 amplicons span respectively the region between exons 1 and 6 and the region between exons 5 and 10. L = 100 basepair DNA ladder (Fermentas).

The studied series were screened for the presence of splicing events by RT-PCR. Two overlapping fragments of *MEFV *cDNA, one from exon 1 to 6, and one from exon 5 to 10, were amplified. Normal transcripts were detected, in addition to the already described transcript, *MEFV*-d2, that was variously expressed (Figure 1).

**Figure 2:**
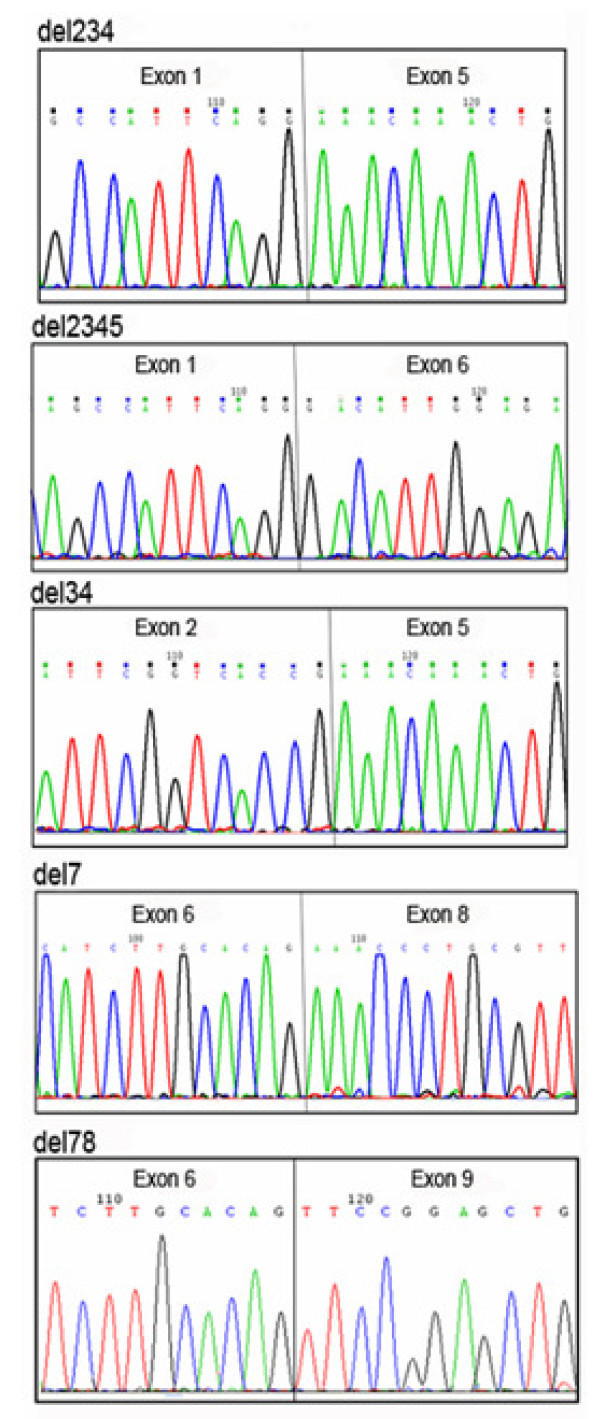
**Sequencing chromatograms showing the deletion junctions of the 5 novel splicing events**.

Five novel alternative splicing events were identified in both patients and controls. Three of them were observed from the 5' PCR products which lacked either exons 2 to 4 (del234), exons 2 to 5 (del2345) or exons 3 and 4 (del34) (Figure 1, left panel). Two other alternative splicing events were identified in the 3' fragment, one lacking exon 7 (del7), and one lacking exons 7 and 8 (del78) (Figure 1, right panel). The deletion boundaries were characterized by sequencing (Figure 2). This revealed that the splicing events derived from the *MEFV *canonical splice-sites.

The distribution of the 5 new splicing events (Table 1) showed that del7 and del78 were always found together in a given individual. del234, del7 and del78 were found in the majority of the individuals. No correlation was observed between the new alternative splicing events and the *MEFV *exonic mutations, and no significant difference was elicited between the genetically confirmed FMF patients, the apparently heterozygous patients and the healthy individuals.

## Discussion

The detection of only one *MEFV *mutation in clinically diagnosed FMF patients and the highly variable phenotype among FMF patients (even those having the same *MEFV *genotype) have always been a subject of concern. Modifying genes could be a possible explanation for such observations, as well as the fact that only one *MEFV *mutation may cause the disease that could be dominant with a variable expressivity. Diseases which pattern of inheritance could be recessive in some families, and dominant in others have already been described [16]. This work aimed at investigating the expression pattern of the *MEFV *gene and trying to relate it to the clinical picture of the patients.

The study of *MEFV *transcripts in clinically diagnosed FMF patients having one or two *MEFV *mutations and in healthy controls revealed 5 novel exon skipping events (del234, del2345, del34, del7 and del78) (Figures 1 and 2), and one previously reported one (*MEFV*-d2) [12]. Skipping of exons 2 to 4, 7 and 7-8 were the most frequently observed events (Table 1).

Molecular events that could cause these alternative spliced transcripts are still unclear. Sequencing of introns 6 and 7 did not show any relevant sequence variation in the tested individuals. Furthermore, the absence of correlation between the *MEFV *point mutations harboured by the patients and controls, and the observed transcripts does not support impairment of splicing regulatory elements that would result in exon skipping [17]. The selective presence of these transcripts among individuals, suggests they might be due to modifying genes or to some external factor such as a reaction to an allergen.

The presence of these novel alternative splicing events in both controls and FMF patients' PBLs seems not in favour of a causal effect of these transcripts in the disease pathogenesis. Previous quantitative studies aiming at correlating disease and *MEFV *mRNA expression showed inconsistent results. *MEFV *transcript levels decreased significantly in FMF patients as compared to controls in the initial reports [18,19], but no significant difference was evidenced in a more recent series [20]. The present study opens a new trail to be addressed regarding the FMF pathophysiology in that the patient's phenotype could be modulated by variations of the different transcripts' ratio. Such a molecular mechanism has been recently described in cystic fibrosis patients who displayed altered regulation of Toll-like Receptor-4 splice variants [21]. A larger series is warranted to replicate our findings. Further analyses of the relative amounts of each transcript are also necessary to confirm or rule out their role in the pathogenic mechanisms underlying inflammation in FMF patients.

## Conclusions

In conclusion, we identified 5 novel *MEFV *splicing events observed in both clinically diagnosed FMF patients and controls. The observation of such qualitative variability in the expression of the *MEFV *gene suggests that this gene is subjected to a complex transcriptional and post-transcriptional regulation. The relative production of the different transcripts is a possibility that could modulate the physiopathological aspect of the disease.

## Competing interests

The authors declare that they have no competing interests.

## Authors' contributions

MMH designed the project and wrote the article. NN looked for the patients and performed the RNA extraction and the RT-PCR followed by polyacrylamide gel electrophoresis and sequencing. EC helped in the results analysis, NJ assisted and participated in the experimental work, and AM directed and supervised the study.

## Pre-publication history

The pre-publication history for this paper can be accessed here:

http://www.biomedcentral.com/1471-2350/11/87/prepub
